# Mutant p53 drives the loss of heterozygosity by the upregulation of Nek2 in breast cancer cells

**DOI:** 10.1186/s13058-020-01370-y

**Published:** 2020-12-02

**Authors:** Amr Ghaleb, Malik Padellan, Natalia Marchenko

**Affiliations:** 1 Department of Pathology, Stony Brook University, Stony Brook, NY 11794-8691 USA; 2 Biologics Process Research & Development, Merck & Co., Inc., Kenilworth, NJ 07033 USA

**Keywords:** Mutant p53, Nek2, LOH, Centrosome clustering, Breast cancer

## Abstract

**Background:**

Mutations in one allele of the TP53 gene in early stages are frequently followed by the loss of the remaining wild-type p53 (wtp53) allele (p53LOH) during tumor progression. Despite the strong notion of p53LOH as a critical step in tumor progression, its oncogenic outcomes that facilitate the selective pressure for p53LOH occurrence were not elucidated.

**Methods:**

Using MMTV;ErbB2 mouse model of breast cancer carrying heterozygous R172H p53 mutation, we identified a novel gain-of-function (GOF) activity of mutant p53 (mutp53): the exacerbated loss of wtp53 allele in response to γ-irradiation.

**Results:**

As consequences of p53LOH in mutp53 heterozygous cells, we observed profound stabilization of mutp53 protein, the loss of p21 expression, the abrogation of G2/M checkpoint, chromosomal instability, centrosome amplification, and transcriptional upregulation of mitotic kinase Nek2 (a member of Never in Mitosis (NIMA) Kinases family) involved in the regulation of centrosome function. To avoid the mitotic catastrophe in the absence of G2/M checkpoint, cells with centrosome amplification adapt Nek2-mediated centrosomes clustering as pro-survival mutp53 GOF mechanism enabling unrestricted proliferation and clonal expansion of cells with p53LOH. Thus, the clonal dominance of mutp53 cells with p53LOH may represent the mechanism of irradiation-induced p53LOH. We show that pharmacological and genetic ablation of Nek2 decreases centrosome clustering and viability of specifically mutp53 cells with p53LOH.

**Conclusion:**

In a heterogeneous tumor population, Nek2 inhibition may alter the selective pressure for p53LOH by contraction of the mutp53 population with p53LOH, thus, preventing the outgrowth of genetically unstable, more aggressive cells.

## Introduction

P53 is a tumor suppressor that plays a crucial role in inducing cancer cell death and growth arrest to protect the genome from the accumulation of DNA errors in response to genotoxic stress [1]. TP53 is the most frequently mutated gene in human breast cancer and, particularly, in Her2(ErbB2)-positive breast cancer (72%), where it is associated with poor outcomes for patients [2]. Typically, mutations in the TP53 gene occur through a two-hit mechanism, where a missense mutation in one allele is followed by loss of the remaining wtp53 allele (p53LOH, loss of heterozygosity). Markedly, the frequency of p53LOH increases as cancer progress: 52% of stage 1, but only 20% of stage 2 breast cancer patients retain wild type p53 (wtp53) allele [3], that suggests the strong selective pressure for p53LOH occurrence during tumor progression. It is generally accepted that p53LOH is a crucial oncogenic event in tumorigenesis. However, understanding the precise mechanism and biological outcomes of p53LOH has been hindered by the lack of relevant experimental in vitro models. Nevertheless, it becomes an important clinical question as the targeting of p53LOH occurrence may lead to novel therapeutic strategies that delay or hinder tumor progression. Hence, we sought to elucidate the functional outcomes of p53LOH that may generate the selective pressure for the loss of wtp53 allele during tumor progression in mutant p53 (mutp53) heterozygous mammary tumors leading to the expansion of cells with p53LOH.

In our previous study, we established and characterized a novel MMTV;ErbB2 mouse model carrying both wtp53 and R172H mutp53 alleles (heterozygous mice, H/+;ErbB2 after that) that mimics early stages of Her2-positive breast cancer [3]. We identified a novel oncogenic activity of mutp53: the exacerbated loss of wtp53 allele in response to irradiation compared to p53−/+;ErbB2 mice. We found that p53LOH is associated with the marked stabilization of mutp53 protein in vivo and in vitro, enhanced chromosomal aberrations, and increased metastases only in the presence of mutp53 allele [3]. As the elevated level of mutp53 protein has been proposed to be essential for its oncogenic activities [4, 5], p53LOH with subsequent stabilization of mutp53 protein may represent key tumor-promoting steps in vivo*.* Nevertheless, it remains to be elucidated how mutp53 aggravates p53LOH and metastases in response to genotoxic stress such as γ-irradiation.

The previous ectopic expression studies suggested that in heterozygous cells, mutp53 may exert its oncogenic activities via the dominant-negative (DN) mechanism by inhibiting the tumor-suppressive function of wtp53 allele or in the gain-of-function (GOF) manner [6, 7]. To evaluate the interplay between endogenous wtp53 and mutp53 in heterozygosity, we generated cell lines from mammary tumors of heterozygous mice with an identical genetic background. Surprisingly, despite a strong notion of the mutp53 DN effect, we have not observed the global suppression of “canonical” wtp53 target genes such as p21, sestrins, and Mdm2 in response to irradiation in the presence of mutp53 allele [3]. Consistent with these findings, here we demonstrate that wtp53 allele in mutp53 heterozygous cells (H/+;ErbB2) is competent partially to induce G2/M checkpoint and growth arrest in response to irradiation. Conversely, p53LOH (H/−; ErbB2 cells) completely abrogate G2/M checkpoint and sustain the S-phase after irradiation leading to cell cycle re-entry with genomic aberrations. Therefore, the competitive growth advantage of cells with p53LOH over mutp53 heterozygous cells may underlie the exacerbated p53LOH, which we observed in vivo. We hypothesized that irradiation-induced p53LOH generates the clonal pool of genetically unstable cells prone to expand after DNA damage, leading to tumor progression and metastases.

Here, we aimed to identify potential vulnerabilities of cells with p53LOH that would provide a therapeutic opportunity to prevent the expansion of cells with p53LOH. The transcriptional and functional characterization of cell lines with distinct p53 deficiencies identified Nek2 (a member of Never in Mitosis (NIMA) Related Kinases family) as a potential target for p53LOH prevention. We demonstrated that the presence of functional wtp53 allele reduces sensitivity to specific Nek2 inhibitor JH295, while p53LOH significantly sensitizes cancer cells to Nek2 inhibition and prevents p53LOH occurrence after irradiation. Hence, our data suggest targeting Nek2 as the potential strategy to avoid p53LOH onset in the context of γ-radiation.

## Materials and methods

### Metabric data

Human Metabric data analysis, of the somatic mutation profiles of 2433 breast cancers, was done using data from a retrospective study [8]. The data is deposited and is publicly available at http://www.cbioportal.org. The analysis was done using the program and tools made available online at http://www.cbioportal.org.

### Mice

MMTV-ErbB2 mice carrying activated ErbB2 (strain FVBN-Tg(MMTV-ErbB2)NK1Mul/J) were from Jackson Labs. p53 R172H (called p53H/H) and control p53 null (p53−/−) mice (C57Bl6J background) were a gift from G. Lozano [9]. p53H/−;ErbB2 mice were generated by crossing ErbB2 mice with p53−/− mice and then breeding the p53+/−;ErbB2 progeny with p53H/H mice. p53H/−;ErbB2 mice were then crossed to generate p53H/H;ErbB2 and p53−/−;ErbB2 females for analysis. p53+/+;ErbB2 were generated from crossing of p53H/+;ErbB2 and p53+/−;ErbB2 mice. Mice carrying the floxed p53R248Q mutation (referred to as floxQ) was generated as described before [10]. For all mice genotypes, only female littermates were used for all analyses. Animals were monitored weekly to determine their breast cancer and sarcoma onset and were promptly killed when their tumors reached 4 cm^3^ in volume or when animals appeared moribund. Careful necropsies were performed, and tumors and all major organs collected, fixed in 10% formalin, embedded in paraffin, and sectioned for histopathologic analysis. For survival analysis, *P* values were determined by log-rank analysis. Mice were treated according to guidelines approved by the Institutional Animal Care and Use Committee at Stony Brook University.

### Cell lines

Human ErbB2-positive breast cancer cell lines ZR-75-30 carrying wild type TP53, and BT474, SKBR3, carrying E285K, R175H *TP53* mutations respectively, were purchased from ATCC. Establishing mouse mammary tumors cell lines was described before [11]. Mouse mammary tumor cell lines: p53+/+;ErbB2, p53H/+;ErbB2, and p53−/+;ErbB2 were isolated from their corresponding mammary tumors and maintained in culture. P53H/−;ErbB2 cells were obtained from p53H/+;ErbB2 tumors with confirmed LOH and p53−/−;ErbB2 cells were obtained from p53−/+;ErbB2 tumors with confirmed LOH. Where shown, cells were treated with 1.2 μM of Nek2 specific inhibitor JH295 (Tocris Bioscience).

### Gamma irradiation

For γ-irradiation of cells, a 137Cs source with a dose rate of 0.8 Gy/min was used, for a total of 0.1 Gy or 9 Gy. Non-irradiated cells (sham) were placed in the room without being exposed to irradiation.

### Immunofluorescence

For IF on cells, media was aspirated from cells grown on chamber slides, cells were fixed with methanol at − 20 °C for 10 min, and then washed 3x with PBS. Cells were permeabilized with 0.2% Tween 20 in PBS at RT for 10 min, and then incubated with blocking buffer [10% normal horse serum (NHS) and 0.1% Tween 20 in PBS], for 1 h at 37 °C. Cells were then stained with rabbit anti-γtubulin (1:200, Sigma) for 1 h at 37 °C, and then washed 3x with PBS. Goat anti-rabbit Alexa fluor-labeled 568 secondary antibodies (Molecular Probes) at 1:500 dilution for 30 min at 37 °C, counterstained with Hoechst 33258 (2 μg/ml), mounted with Prolong gold (Molecular Probes), and cover-slipped. Images were acquired at × 600 total magnification using a Nikon Eclipse Ti-S microscope (Nikon Instruments) equipped with QI-Click camera (QImaging). Where applicable, quantification of centrosome number was performed on 10 images of randomly selected fields per genotype per treatment.

### Determination of LOH of the *p53+* locus

Mouse p53H/+;ErbB2 cells were irradiated (9 Gy), or not, and Nek2 inhibitor was added (1.2 μg/ml) 6 h post irradiation. Cells were maintained in culture for 10 days with or without Nek2 inhibitor and fresh media, with or without Nek2 inhibitor, was replenished every 3–4 days. DNA was then extracted using QIAmp DNA Micro Kit (Qiagen). An equal amount of DNA was used for PCR amplification of p53 locus using primers described before [9]. An equal volume of the amplified product was electrophoresed through a 1.5% agarose gel. Amplified DNA bands were visualized, and the image captured using FluoroChem HD2 (ProteinSimple). LOH was determined based on the presence or absence of the amplified wild type band. Band intensity of wtp53 and mutp53 amplicons were measured using ImageJ [12].

### Real-time PCR

All real-time PCR was done on cultured cells using 3 biological replicas. For determination mRNA transcript level, RNA was extracted from cultured cells using Trizol as per manufacturer instructions. For cDNA synthesis, 200 ng/sample was used in a 20 μl reaction volume prepared from QuantiTect Reverse Transcription Kit (Qiagen). After cDNA synthesis, the reaction volume was diluted to 200 μl using DEPC-treated water. For qPCR, 1 μl of the diluted cDNA was used per reaction volume. The following primers were used: for human cell lines: NEK2 [F] 5′-AGCGAGCTCTCAAAGCAAGA-3′, [R] 5′-ACTGAGGATGGAAGATTAAGAAGT-3′; HPRT [13] [F] 5′-GCTATAAATTCTTTGCTGACCTGCTG-3′, [R] 5′-AATTACTTTTATGTCCCCTGTTGACTGG-3′; for mouse cell lines: cyclin E2 [F] 5′-ATGTCAAGACGCAGCCGTTTA-3′, [R] 5′-GCTGATTCCTCCAGACAGTACA-3′; Nek2 [F] 5′-TAACGGGATGCGTATGGCAG-3′, [R] 5′-TTAACTGGCACAGTGAGCGT-3′; Hprt [14] [F] 5′-GGCTATAAGTTCTTTGCTGACC-3′, [R] 5′-CTCCACCAATAACTTTTATGTCC-3′. For all real-time PCR, amplification was done using Quantitech sybr green (Qiagen) reaction mixture, and detection was done using QuantStudio3 (Thermo Fisher Scientific).

### siRNA and CRISPR/Cas9 treatment

Human p53-specific siRNA was purchased from Santa Cruz Biotech. CRISPR/Cas9 was used to delete *p53* or *Nek2* from cultured mouse cell lines by transfecting cells with *p53* or *Nek2* double nickase plasmid (Santa Cruz Biotech), using TransIT-X2® Transfection Reagent (Mirusbio) according to manufacturer’s recommendations. Selection of transfected cells was done by adding puromycin (6 μg/ml) (Fisher) to culture media 2 days post transfection. Selected cells were maintained in media with puromycin (6 μg/ml) throughout.

### Cell cycle analysis

Cultured cells were harvested by trypsinization and pelleted by spinning at 1500 r.p.m. for 10 min. The cell pellet was washed twice with PBS, then fixed in 70% ethanol. The cells were pipetted gently up and down to loosen the cells in a suspension and stored in − 20 °C overnight. The cells were then pelleted by spinning at 1500 r.p.m. for 10 min, washed once in PBS, then resuspended in permeabilization buffer (0.25% tritonX100 in PBS) and incubated for 15 min at RT. The cells were then pelleted and resuspended in staining solution (20 μg/ml propidium iodide and 10 μg/ml RNase A in PBS) and incubated in the dark on ice for 30 min before analysis. Cell cycle analysis by flow cytometry was done at Stony Brook Flowcytometry Core Facility, using Becton Dickinson FACSCAN analyzer.

### Immunoblot analysis

For immunoblots, cell lysates with equal total protein content (2–20 μg) were blotted with antibodies to p53 (FL393), p21 GAPDH, Hsc70, Nek2, and tubulin (all from Santa Cruz Biotechnology); γH2AX (all from Cell Signaling)). All immunoblots were repeated at least two times.

### Colony formation assay and staining

Mouse cells were plated at 20 × 10^3^ cells per well and treated, or not, with Nek2 inhibitor (1.2 μg/ml). Cells were maintained in culture for 15 days with or without Nek2 inhibitor and fresh media, with or without Nek2 inhibitor, was replenished every 3–4 days. For staining, cells were fixed by adding 4% paraformaldehyde to growth media (final concentration 1%) for 10 min at RT. The media was then aspirated, and a staining solution (0.5% crystal violet in 20% methanol) was gently added to cover wells. Cells were left to stain for 20 min at RT, and the staining solution was removed by aspiration. Excess staining was then washed by gently dipping one plate at a time into a beaker of water. The plates were then air-dried and visualized.

### Purification and preparation of RNA for microarray expression analysis

RNA was processed from cells that had reached 80–90 confluency. Total RNA from cultured cells was extracted using Trizol reagent as recommended by the manufacturer (Invitrogen; Carlsbad, CA). RNA was subjected to DNase I treatment in order to remove any contaminating genomic DNA. Final purification was performed on RNAeasy columns (Qiagen; Valencia, CA), according to the manufacturer’s recommendations. The integrity, quality, and quantity of total RNA were confirmed by Eukaryotic Total RNA Nano Bioanalyzer (Agilent Technologies) at the genomic core facility at Stony Brook University. RNA with OD260/OD280 > 1.8 and RNA integrity number of > 8.5 were submitted for microarray analysis.

### Microarray expression analysis

After the QC procedures, mRNA from eukaryotic organisms was enriched using oligo(dT) beads. For prokaryotic samples, rRNA was removed using a specialized kit that cleaves the mRNA. The mRNA was then fragmented randomly in a fragmentation buffer, followed by cDNA synthesis using random hexamers and reverse transcriptase. After first-strand synthesis, a custom second-strand synthesis buffer (Illumina) was added with dNTPs, RNase H, and *Escherichia coli* polymerase I to generate the second strand by nick-translation. The final cDNA library was used for a round of purification, terminal repair, A-tailing, ligation of sequencing adapters, size selection, and PCR enrichment. Library concentration was first quantified using a Qubit 2.0 fluorometer (Life Technologies) and then diluted to 1 ng/μl before checking insert size on an Agilent 2100 and quantifying to greater accuracy by quantitative PCR (Q-PCR) (library activity > 2 nM).

The original raw data from Illumina HiSeqTM are transformed into Sequenced Reads by base calling. Raw reads are filtered to remove reads containing adapters or reads of low quality so that downstream analyses are based on clean reads. TopHat2 software was used for mapping sequences of animal genome. The mismatch parameter is set to two, and other parameters are set to default. Only filtered reads are used to analyze the mapping status of RNA-seq data to the reference genome.

Gene expression level was measured by transcript abundance. The gene expression level was estimated by counting the reads that map to genes or exons. Read count was proportional to the actual gene expression level and to the gene length and the sequencing depth. The FPKM (Fragments Per Kilobase of transcript sequence per Millions base pairs sequenced) was used in order for the gene expression levels estimated from different genes and experiments to be comparable. HTSeq software was used to analyze the gene expression levels using the union mode. The result files present the number of genes with different expression levels and the expression level of single genes. In general, an FPKM value of 0.1 or 1 is set as the threshold for determining whether the gene is expressed or not. The overall results of FPKM cluster analysis were done using the log10(FPKM+ 1) value.

Centrosome proteins and their genes were identified based on data from MiCroKit database [15] (http://microkit.biocuckoo.org/). FPKM values of these identified genes were used for cluster analysis using the publicly available program, Morpheus (https://software.broadinstitute.org/morpheus/).

### Statistics and reproducibility

All statistical analysis between groups was done using the *t* test. Significance was determined at *p* < 0.05. Cell culture experiments were repeated three times.

## Results

### p53LOH enhances cell proliferation

**Fig. 1:**
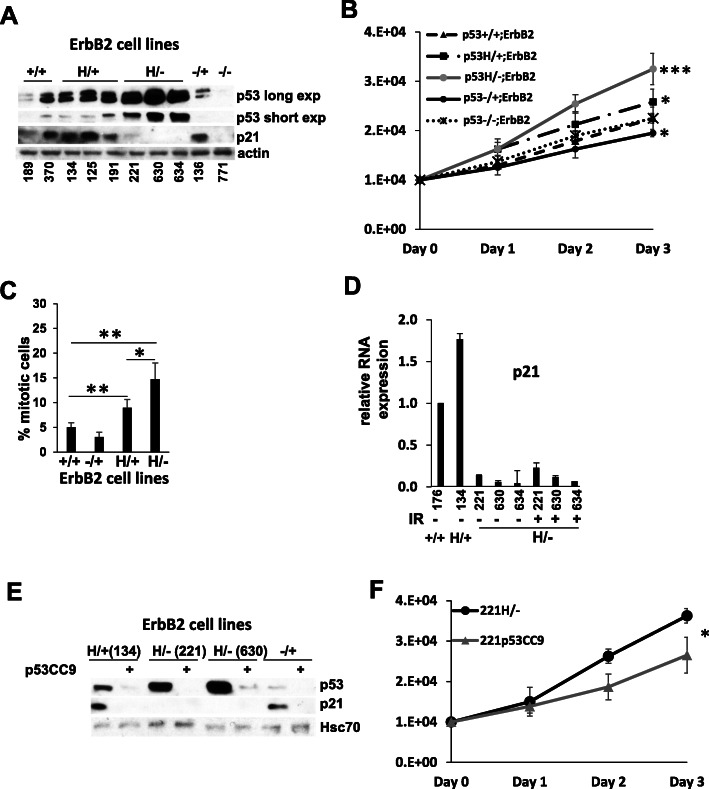
Mutp53 promotes cell proliferation. **a** Western blot analysis of p53 and p21 levels in mouse ErbB2 mammary epithelial tumor cell lines with different p53 status. Actin is loading control. **b** Growth curve of mouse ErbB2 mammary epithelial tumor cell lines with different p53 status. *n* = 3 independent experiments per genotype (one cell line per genotype except for p53+/+ and p53 H/− where 2 different cell lines derived from different tumors and result per genotype was averaged). **c** Bar graph showing percent mitotic cells in mouse ErbB2 mammary epithelial tumor cell lines with different p53 status. Each bar represents the average percent of mitotic per genotype counted from at least 5 randomly selected fields at × 400 magnification (one cell line per genotype except for p53+/+ and p53 H/− where 2 different cell lines derived from different tumors and result per genotype was averaged). **d** Bar graph showing relative mRNA expression level of p21 in ErbB2 mammary epithelial tumor cell lines with different p53 status. *n* = 3 independent experiments per cell line per genotype. **e** Western blot analysis of p53 and p21 levels in mouse ErbB2 mammary epithelial tumor cell lines with different p53 status before and after CRISPR/Cas9 p53 deletion (p53CC9). Hsc70 is loading control. **f** Growth curve of mouse ErbB2 mammary epithelial tumor cell line with mutp53, before and after CRISPR/Cas9 p53 deletion (p53CC9). *n* = 3 independent experiments per cell line. Where applicable *p < 0.05; **p < 0.01; ***p < 0.001. Error bars represent ± SD

Previously, we established a novel genetic mouse model of early stages of Her2 positive breast cancer by crossing MMTV-ErbB2 and R172H KI mice [16]. We characterized the following mouse genotypes: R172H/wtp53;ErbB2 (H/+;ErbB2), p53null/wtp53;ErbB2 (−/+;ErbB2), and wtp53/wtp53;ErbB2 (+/+;ErbB2). The murine R172H p53 mutation corresponds to human R175H p53 mutation, which was identified as a hotspot in ErbB2 breast cancer [17]. To evaluate the phenotypic effects of mutp53 in heterozygosity, we established stable cell lines from mouse mammary tumors of +/+;ErbB2, H/+;ErbB2, H/−;ErbB2 (R172H/p53null;ErbB2), −/+;ErbB2, and −/−;ErbB2 genotype (three biological replicas per genotype) (Fig. 1a). In contrast to the existing human breast cancer cell lines that are mutp53 homo- or hemizygous, our panel of cell lines (isogenic and non-isogenic) allows us to evaluate the pathological consequences of p53LOH in the well-controlled model.

We found that compared to p53+/+;ErbB2 and p53−/+;ErbB2 cells, the presence of mutp53 allele in heterozygous cells elevates the total p53 protein level, while p53LOH leads to further stabilization of mutp53 protein (Fig. 1a). We have previously shown that γ-irradiation leads to the profound loss of wtp53 allele in p53H/+;ErbB2, but not in p53−/+;ErbB2 cell lines [3]. Hence, we utilized the established cell line panel to elucidate the mechanism of mutp53-mediated p53LOH.

**Fig. 2:**
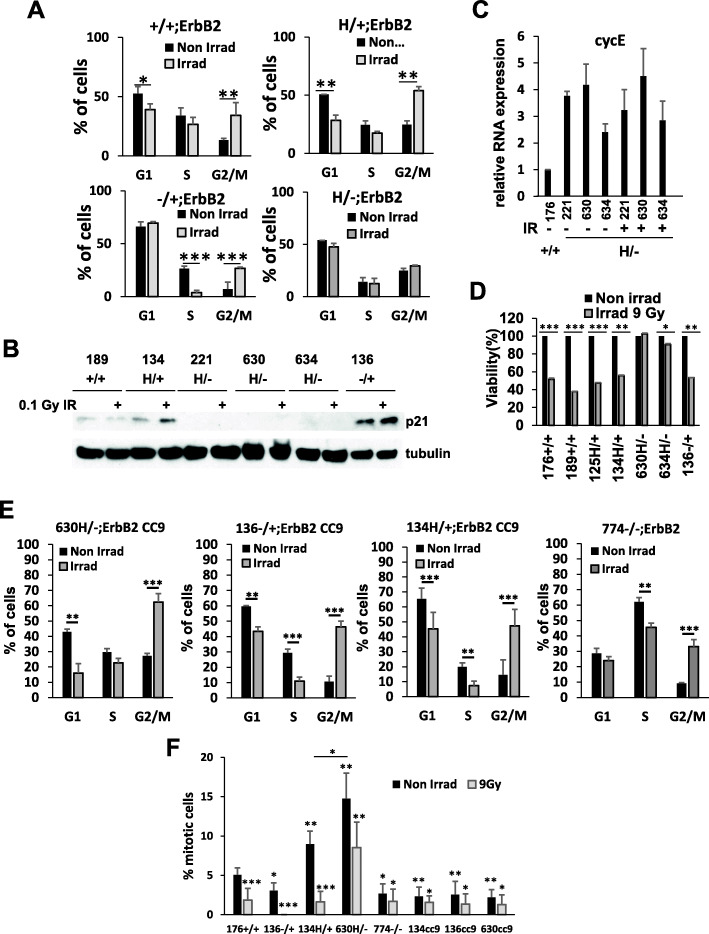
Mutp53 suppresses cell cycle checkpoint following γ-irradiation. **a** Aberrant cell cycle checkpoint following γ-irradiation in p53H/−;ErbB2 cells. Bar graphs showing cell cycle analysis of p53+/+;ErbB2, p53H/+;ErbB2, p53−/+;ErbB2, and p53H/−;ErbB2 cell lines irradiated (gray bars) or not (black bars). *n* = 3 independent experiments per genotype (one cell line per genotype except for p53+/+ and p53 H/− where 2 different cell lines derived from different tumors and result per genotype was averaged). **b** Western blot analysis of p21 level before and 24 h after γ-irradiation (0.1 Gy) in mouse ErbB2 mammary epithelial tumor cell lines with different p53 status. α-Tubulin is loading control. **c** Bar graph showing relative mRNA expression level of CycE before and 24 h after γ-irradiation in ErbB2 mammary epithelial tumor cell lines with different p53 status. *n* = 3 independent experiments per cell line per genotype. **d** Bar graph showing percent viability before and 24 h after γ-irradiation in ErbB2 mammary epithelial tumor cell lines with different p53 status. *n* = 3 independent experiments per cell line per genotype. **e** Restoration of cell cycle checkpoint 24 h post γ-irradiation p53-null cells. Bar graphs showing cell cycle analysis of p53H/−;ErbB2, p53H/+;ErbB2, p53−/+;ErbB2 following CRISPR/Cas9 p53 deletion (p53CC9) and in p53−/−;ErbB2 cell lines irradiated (gray bars) or not (black bars). *n* = 3 independent experiments per genotype. **f** Bar graphs showing mitotic index in different cell lines irradiated (gray bars) or not (black bars) (result for each irradiated genotype was compared to its own control). *n* = 3 independent experiments per genotype. Where applicable *p < 0.05; **p < 0.01; ***p < 0.001. Error bars represent ± SD

Markedly, the cell growth analysis demonstrated that p53 LOH (H/−;ErbB2 cells) increases cell proliferation over cells with wtp53 allele (+/+;ErbB2, H/+;ErbB2 and −/+;ErbB2 cells) and over cells null for p53 (p53−/−;ErbB2) (Fig. 1b). Consistent with growth curves, loss of wtp53 allele in mutp53 heterozygous cells (H/−;ErbB2) shows the highest percentage of cells in mitosis compared to other p53 genotypes (Fig. 1c). Our previous study demonstrated that in H/+;ErbB2 cells, mutp53 does not exert a global DN effect over wtp53 allele in response to DNA damage [3]. In agreement with previous data, here we show that the presence of wtp53 allele in H/+;ErbB2 cells is sufficient to induce canonical p53 target p21 at the RNA (Fig. 1d) and protein level (Figs. 1a, 2b) under normal conditions. Loss of wtp53 allele in H/−;ErbB2 and p53−/−;ErbB2 cells abrogates p21 expression (Fig. 1d), which remains undetectable even after irradiation (Fig. 2b). Consistent with the transcriptional activity of wtp53 in heterozygous cells, CRISPR/Cas9-deletion of p53 (mutp53 and wtp53) obliterates the basal p21 expression in unstressed H/+;ErbB2 cells (Fig. 1e).

This finding suggests that the loss of wtp53-mediated p21 expression may enhance proliferation and provide a competitive advantage to cells with p53LOH over cells retaining wtp53 allele. Additionally, CRISPR/Cas9 deletion of mutp53 in H/−;ErbB2 cells decreased cell proliferation significantly (Fig 1f), suggesting that mutp53 enhances cell proliferation in GOF manner.

These results led us to speculate that under normal conditions, spontaneous p53LOH in heterogeneous H/+;ErbB2 tumor population provides a competitive growth advantage to H/−;ErbB2 cells by two complementary mechanisms: the ablation of basal p21 expression via loss-of-function mechanism and stabilization of mutp53 protein enabling its GOF activities.

### p53LOH abrogates the G2/M checkpoint after irradiation

An increased incidence of p53LOH in the presence of mutp53 allele after irradiation [3] set us to investigate the mechanism by which mutp53 promotes p53LOH. The cell cycle analysis demonstrated that p53LOH in mutp53 cells abrogates G2/M checkpoint, which is preserved in the presence of wtp53 allele in −/+;ErbB2 and is partially functional in H/+;ErbB2 (Fig. 2a). As p21 was shown to play a distinct role in the G2/M checkpoint [18, 19], we analyzed p21 protein level in response to irradiation. To avoid nonspecific effects of high dose irradiation, in subsequent experiments, we utilized the low dose irradiation (0.1 Gy). Consistent with the transcriptional activity of wtp53 allele in heterozygous cells (Fig. 1d), we found that irradiation induces p21 in H/+;ErbB2 cells, while the loss of wtp53 allele (H/−;ErbB2) correlates with a lack of detectable p21 protein even after irradiation (Fig. 2b). The dominance of the wtp53 allele over mutp53 in response to DNA damage and the induction of p21 has been reported previously [20].

Cyclin E is necessary for centrosome duplication in the S phase that precedes the G2/M transition [21]. Previously, we demonstrated a significant reduction of cyclin E2 transcription after irradiation in the presence of wtp53 allele (p53H/+;ErbB2 and p53−/+;ErbB2), which is indicative of G2/M arrest [3]. Contrary, irradiation does not affect cyclin E2 transcription in H/−;ErbB2 (Fig. 2c) that was associated with the deficient G2/M checkpoint after irradiation (Fig. 2a). In agreement with defective G2/M checkpoint (Fig. 2a), the lack of p21 expression (Figs. 1d and 2bb), and elevated cyclin E2 mRNA (Fig. 2c), H/−;ErbB2 cells sustain proliferation after irradiation (Fig. 2a, d). This is in the stark contrast to continuous growth arrest of −/+;ErbB2 and H/+;ErbB2 cells after irradiation (Fig. 2a, d).

Importantly, we found that mutp53 CRISPR/Cas9 deletion in H/−;ErbB2 cells restored G2/M arrest after irradiation, as indicated by increased G2/M populations (Fig. 2e). A similar cell cycle profile was observed in H/+;ErbB2 and −/+;ErbB2 cells after p53 CRISPR/Cas9 deletion (Fig. 2e). Consistently, p53−/−;ErbB2 cells maintain functional G2/M checkpoint as indicated by increased G2/M population after irradiation (Fig. 2e), with no mitotic slippage except in H/−;ErbB2 cells (Fig. 2f). Of note, the cell cycle profiles of H/−;CC9 and −/−;ErbB2 cells are slightly different. The −/−;ErbB2 line was established from −/+;ErbB2 tumor that lost its wtp53 allele through LOH, while the H/−;CC9 cells had mutp53 before CRIPSR depletion. The original presence of mutp53 in the H/−;ErbB2 cells may have led to genetic alterations that are persistent after p53 deletion leading to the differences in the cell cycle profile observed in H/−;CC9 line and −/−;ErbB2 cells. Most importantly, all CC9 (including 630H/−;CC9) and −/−;ErbB2 cells exhibit functional G2/M checkpoint post-irradiation. This data indicates wtp53 independent G2/M checkpoint; however, skipping the G2/M arrest is driven by mutp53 (Fig. 2a). These results strongly suggest that p53LOH in mutp53 heterozygous cells abrogates G2/M checkpoint in the mutp53 GOF manner leading to cell cycle progression after γ-irradiation in the presence of unrepaired DNA (Fig. 2e).

Together, our data indicate that γ-irradiation enhances the clonal expansion of mutp53 cells with p53LOH by providing the competitive growth advantage over cells retaining the wtp53 allele, which induce p21 and undergo G2/M arrest in response to irradiation. Therefore, the clonal dominance of cells with p53LOH may represent the mechanism of irradiation-induced p53LOH.

### p53LOH drives chromosomal instability in mutant p53 cancer cells

**Fig. 3:**
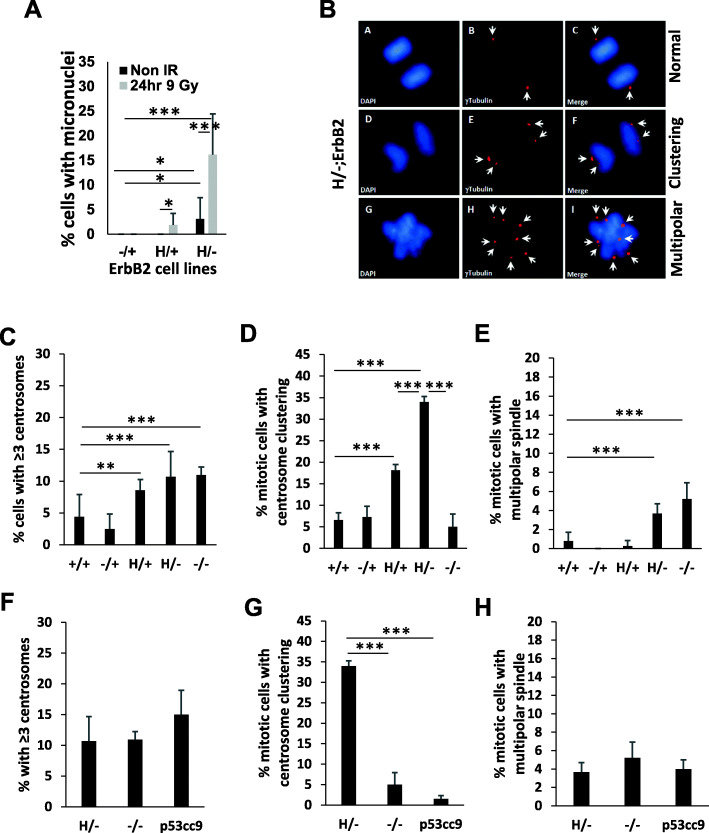
Mutp53 increases centrosomal aberrations and clustering. **a** Bar graph showing percent of cells with micronuclei before and 24 h after γ-irradiation in ErbB2 mammary epithelial tumor cell lines with different p53 status. *n* = 3 independent experiments per cell line per genotype (one cell line per genotype except for p53+/+ and p53 H/− where 2 different cell lines derived from different tumors and p53CC9 3 different cell lines, and result per genotype was averaged). **b** Immunofluorescence staining showing centrosome clustering in mitotic p53H/−;ErbB2-mouse mammary epithelium tumor cell line. Centrosomes identified by γ-tubulin staining (red) and DNA by DAPI (blue). **b** (A–C) Normal bipolar mitosis with one centrosome at each side. **b** (D–F) Bipolar mitosis showing supernumerary centrosome (≥ 3) clustering, 2 centrosomes on each side. **b** (G–I) Multipolar mitosis showing failure of supernumerary centrosomes to cluster. **c**–**e** Bar graphs showing percent of cells with ≥ 3 centrosomes, with centrosome clustering and with multipolar spindle, respectively, in ErbB2 mammary epithelial tumor cell lines with different p53 status. *n* = 3 independent experiments per genotype. **f**–**h** Bar graphs showing percent of cells with ≥ 3 centrosomes, with centrosome clustering and with multipolar spindle, respectively, in p53H/−;ErbB2 cell line before and after CRISPR/Cas9 p53 deletion (p53CC9) and in p53−/−;ErbB2 cell line. *n* = 3 independent experiments per genotype (for **c**–**h**, one cell line per genotype except for p53+/+ and p53 H/− where 2 different cell lines derived from different tumors and p53CC9 3 different cell lines, and result per genotype was averaged). Where applicable *p < 0.05; **p < 0.01; ***p < 0.001. Error bars represent ± SD

While mutp53 was implicated as an essential driver of various forms of chromosomal instability—aneuploidy, translocation, and amplification [22, 23]—the underpinning mechanism of how mutp53 induces chromosomal aberrations remains vague. Previously, we demonstrated that p53LOH in the presence of mutp53 allele is associated with increased chromosomal instability in vivo indicated by the higher incidence of anaphase bridges in mammary tumors [3]. In addition, errors in chromosome segregation (chromosomal instability) during mitosis might be monitored by the formation of micronuclei [24, 25]. Consistent with our previous finding [3], we found that irradiation more profoundly drives chromosomal instability in the presence of a mutp53 allele that is further augmented by p53LOH, as indicated by micronuclei formation (Fig. 3a).

As chromosomal instability may arise from abnormal chromosome segregation in mitosis, we investigated centrosome aberration with respect to p53 status. During mitosis, two centrosomes form spindle poles and direct the formation of bipolar mitotic spindles, which is an essential event for accurate segregation of chromosomes. The presence of more than two centrosomes (centrosome amplification) severely disturbs cytokinesis during mitosis via the formation of more than two spindle poles (Fig. 3b), resulting in an increased frequency of chromosome segregation errors, such as aneuploidy, amplifications, and deletions. These genetic events may further facilitate tumor progression and the acquisition of metastatic phenotype. Significantly, the presence of mutp53 allele in heterozygous cells significantly increases centrosome amplification compared to −/+;ErbB2 cells (Fig. 3b, c) in an apparent DN fashion. Therefore, the elevated centrosome amplification in H/+;ErbB2 cells may increase the incidence of spontaneous p53LOH under normal conditions as compared to −/+;ErbB2 cells. Subsequently, p53LOH (H/−;ErbB2 and −/−;ErbB2 cells) slightly increases the abnormal centrosome number (Fig. 3b, c). On the other hand, the excessive centrosome amplification within tumor cells can be deleterious as it may lead to multipolar mitosis and generate sufficiently high levels of aneuploidy to pose a challenge for cell viability [26]. As a pro-survival mechanism, cancer cells adapt to avoid multipolar mitosis by clustering their extra centrosomes at the two poles of the spindle during mitosis, thus ensuring bipolar chromosome segregation [27]. However, pseudo-bipolar spindle formation through centrosome clustering causes slower mitosis. The latter leads to increased frequency of lagging chromosomes during anaphase and thus to chromosomal instability, thereby explaining the link between supernumerary centrosomes and chromosomal instability [28]. Although centrosome clustering occurs both in vivo [29, 30] and in vitro [31], its underpinning mechanism is not well understood. Thus, we set to determine whether the mutp53 cells ensure cell survival by evasion of multipolar mitosis via centrosome clustering at the expense of chromosomal instability. We observed mitotic cells with centrosome clustering in all mouse mammary tumor cell lines; however, the percent of mitotic cells with centrosome clustering was significantly higher in cells with mutp53 as compared to mitotic +/+;ErbB2, −/+;ErbB2 and −/−;ErbB2 cells (Fig. 3d). Furthermore, p53LOH (H/−;ErbB2 cells) significantly increased mitotic centrosome clustering compared to H/+;ErbB2 cells (Fig. 3d). Notably, the loss of protective wtp53 allele (−/−;ErbB2 and H/−;ErbB2) significantly elevated multipolar mitosis (Fig. 3e), but only H/−;ErbB2 cells adapt centrosome clustering as a pro-survival mechanism to avoid cell death due to mitotic catastrophe (Fig. 3d). In support of GOF mechanism of centrosome clustering, deletion of mutp53 by CRISPR/Cas9 significantly reduced centrosome clustering but does not affect centrosome amplification or multipolar spindle formation Fig. 3f–h.

Together, our data identify centrosome clustering a novel pro-survival GOF mechanism that underlies an increased fitness of mutp53 cancer cells with p53LOH at the expense of chromosomal instability.

### Mutant p53 allele is associated with the elevated Nek2 function

Understanding of how p53LOH enables the proliferation of mutp53 cells (Fig. 1b) and disrupts the mitotic checkpoint (Fig. 2a) in the presence of centrosomal and chromosomal aberrations (Fig. 3) would provide an essential insight into how to prevent the outgrowth of mutp53 cells with p53LOH.

**Fig. 4:**
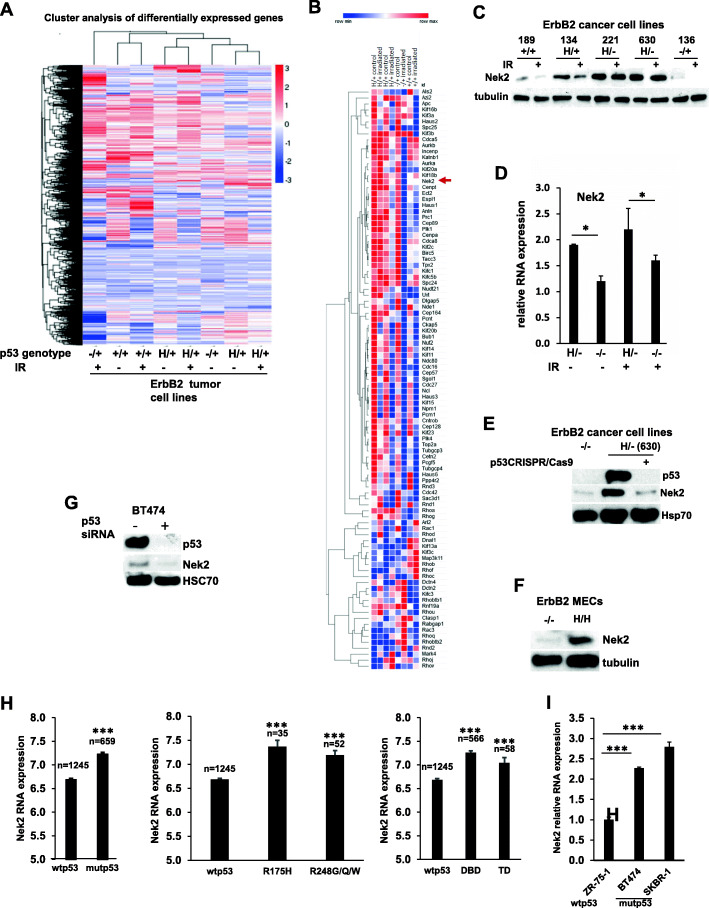
Mutp53 is associated with elevated mRNA and protein levels of NEK2. **a** Heat map showing cluster analysis of differentially expressed genes in p53+/+;ErbB2, p53H/+;ErbB2 and p53−/+;ErbB2 before and after γ-irradiation (9 Gy, 24 h). **b** Heat map of RNAseq analysis showing differentially regulated centrosome proteins in p53+/+;ErbB2, p53H/+;ErbB2 and p53−/+;ErbB2 before and after γ-irradiation (9 Gy, 24 h). Arrowhead indicates Nek2. **c** Western blot analysis of Nek2 level before and 24 h after γ-irradiation in mouse ErbB2 mammary epithelial tumor cell lines with different p53 status. α-tubulin is loading control. **d** Bar graph showing relative mRNA expression level of Nek2 in p53H/−;ErbB2 and p53−/−;ErbB2 cell lines before and after irradiation. *n* = 3 independent experiments per genotype (one cell line per genotype except for p53 H/− where 2 different cell lines derived from different tumors and result was averaged). **e** Western blot analysis of p53 and Nek2 levels in p53−/−;ErbB2 cells and in p53H/−;ErbB2 cells before and after CRISPR/Cas9 p53 deletion. Hsp70 is loading control. **f** Western blot analysis of Nek2 level in p53−/− MECs and p53H/H MECs. α-Tubulin is loading control. **g** Western blot analysis of p53 and Nek2 levels in BT474 cells before and after p53 suppression with siRNA. HSC70 is loading control. **h** Bar graphs showing NEK2 relative mRNA expression in patients with wtp53 (*n* = 1245) compared to with mutp53 (*n* = 659) (all mutations combined vs different types of mutations) (DBD = DNA binding domain; TD = tetramerization domain). **i** Bar graph showing Nek2 relative mRNA expression in human breast cancer cell lines with different p53 status. *n* = 3 independent experiments per genotype. Where applicable *p < 0.05; **p < 0.01; ***p < 0.001. Error bars represent ± SD

To identify the putative mechanism, we performed RNAseq of mouse mammary tumor cell lines with various p53 genotypes, irradiated, or not (Fig. 4a). The expression analysis of genes involved in the regulation of mitosis identified Nek2 among the top 10 differentially up-regulated genes in the presence of mutp53. Neks (Never in Mitosis (NIMA) Kinases) are a family of serine/threonine kinases involved in the regulation of centrosome function and bipolar division during mitosis. Nek2 is overexpressed in various cancers, including Her2 positive breast cancer, where it predicts poor overall survival [32, 33]. RNAseq analysis showed upregulation of Nek2 at basal level in H/+;ErbB2 as compared to +/+;ErbB2 cells (Fig. 4b).

We focused on studying of Nek2 for the following reasons: (*i*) Nek2 plays an indispensable role for the entry into mitosis and G2/M progression, as it is required for centrosome assembly/maintenance, spindle formation, and chromosome segregation [34–37]. (*ii*) Nek2 overexpression promotes centrosome amplification and aneuploidy by disrupting the mitotic checkpoint, leading to malignant transformation [38, 39]. (*iii*) Silencing Nek2 with siRNA inhibited proliferation, induced cell death (due to mitotic errors), and dramatically increased the susceptibility of breast cancer cells to DNA-damaging modalities [38, 39]. (*iv*) Wtp53–Nek2 autoregulatory feedback loop has previously been described [40–42], while no mutp53-Nek2 functional interaction has been investigated. (*v*) Nek2 can be targeted by highly specific small-molecular inhibitor JH29525 that opens the opportunity for therapeutic intervention.

We validated the RNAseq data by Western (Fig. 4c). Consistent with wtp53 as a negative regulator of Nek2 expression [41], we observed the lowest level of Nek2 in +/+;ErbB2 and −/+;ErbB2 cells (Fig. 4c). Furthermore, irradiation downregulates Nek2 in cells carrying at least one p53 allele (Fig. 4c), while the loss of wtp53 allele (H/−;ErbB2) leads to Nek2 upregulation that is insensitive to irradiation on both protein (Fig. 4c) and RNA levels (Fig. 4d). In addition to the loss of wtp53 function, mutp53 in H/−;ErbB2 cells upregulates Nek2 expression in apparent GOF manner as stabilized mutp53 protein in H/−;ErbB2 cancer cells was associated with a higher level of Nek2 mRNA and protein levels compared to −/−;ErbB2 cancer cells (Fig. 4d) or following mutp53 ablation by CRISPR/Cas9 (Fig. 4e). Similarly, mammary epithelial cells (MECs) established from mammary of −/−;ErbB2 mice [17] express significantly lower levels of Nek2 protein compared to H/H;ErbB2 MECs (Fig 4f). Importantly, mutp53 promotes Nek2 expression independently of host and the type of p53 mutation. Mutp53 depletion by siRNA decreases the Nek2 level in human cancer cell line BT474 (E285K) (Fig. 4g).

In further support of the mutp53-Nek2 association in human cancer, a retrospective analysis of the Metabric cohort of breast cancer patients (www.cbioportal.org) demonstrated a significantly higher median of Nek2 mRNA expression in mutp53 patients, regardless of the mutation type, as compared to patients with wtp53 (Fig. 4h). Furthermore, human mutp53 HER2-positive human breast cancer lines (BT474 (E285K), SKBR3 (R175H)) showed significantly higher expression of NEK2 mRNA as compared to ZR-75-1(wtp53) (Fig. 4i).

Together, these experiments indicate that mutp53 can affect Nek2 expression by two complementary mechanisms: the loss of wtp53 inhibitory function and mutp53 GOF upregulation of Nek2. Hence, mutp53-mediated Nek2 expression may reinforce G2/M transition, override G2/M checkpoints, and protect cancer cells from multipolar mitosis at the expense of chromosomal instability.

### Nek2 inhibition prevents p53LOH in mutant p53 heterozygous cells

We hypothesized that deficient checkpoints and the increased proliferation of H/−;ErbB2 cells confer a positive selection for p53LOH during tumor progression. Therefore, the identification of specific vulnerabilities of mutp53 cancer cells with p53LOH would provide the therapeutic opportunity to prevent p53LOH and, thus, the expansion of genetically unstable, more aggressive cancer cells population. As a mutp53-mediated upregulation of Nek2 (Fig. 4) may facilitate G2/M transition by reinforcing centrosome clustering, mutp53 cells with p53LOH may specifically be dependent on Nek2 expression for their survival to avoid multipolar mitosis and mitotic catastrophe.

To test this hypothesis, we investigated the effect of Nek2 inhibitors on mitotic spindle formation and centrosome clustering with respect to p53 genotypes. Several Nek2-specific inhibitors were described in the literature (JH 295, TOCRIS, or TAI-95, Probechem) [43]. In our study, we utilized JH295 (oxindole propynamide, IC50 = 770 nM), which is a highly specific and irreversible Nek2 inhibitor that blocks Nek2 activity via alkylation of residue Cys22, and does not affect the activities of other mitotic kinases (CDK1, PLK1, Aurora B, or Mps1) [43]. Moreover, JH295 does not perturb bipolar spindle assembly or the spindle assembly checkpoint [43]. Given this selective profile, we thought that JH295 is as useful for identifying the biological roles of Nek2 as RNAi interference approach.

**Fig. 5:**
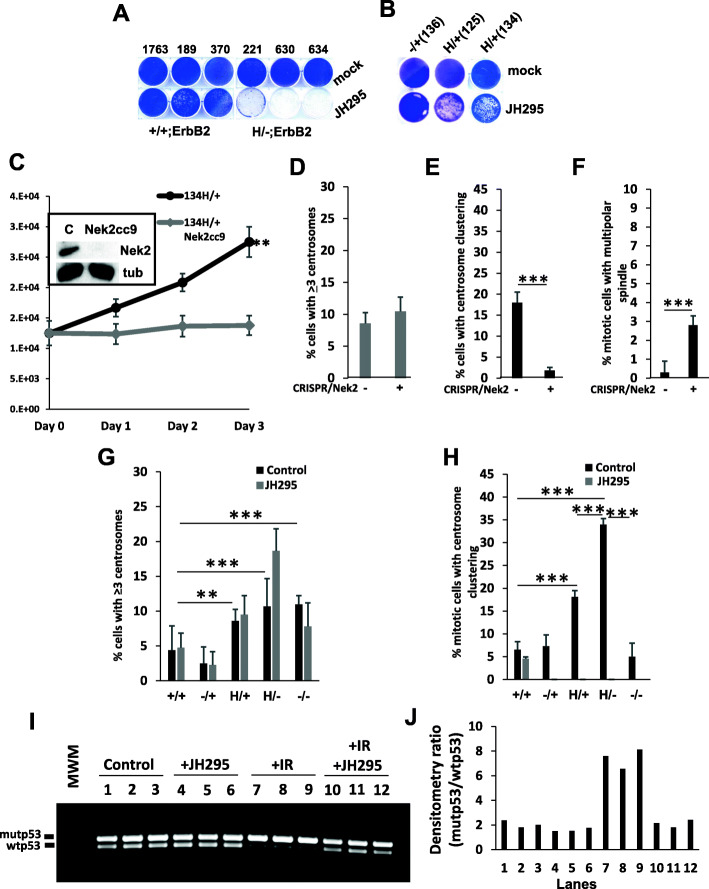
Nek2 ablation suppresses centrosome clustering and p53LOH. **a** Colony formation assay showing suppressed proliferation of H/−;ErbB2 cells, as compared to +/+;ErbB2 cells, following treatment with Nek2 inhibitor JH295. **b** Colony formation assay showing partial suppression of proliferation of H/+;ErbB2 cells, as compared to −/+;ErbB2 cells, following treatment with Nek2 inhibitor JH295. **c** Growth curve showing suppressed proliferation in H/+;ErbB2 cells following CRISPR/Cas9 Nek2 deletion (Nek2cc9). Inset shows western blot for Nek2 before and after CRISPR/Cas9 Nek2 deletion. α-Tubulin is loading control. **d**–**f** Bar graphs showing percent of cells with ≥ 3 centrosomes, with centrosome clustering and with multipolar spindle, respectively, in H/+;ErbB2 cells before and following CRISPR/Cas9 Nek2 deletion. *n* = 3 independent experiments per genotype. **g**, **h** Bar graphs showing percent of cells with ≥ 3 centrosomes and with centrosome clustering, respectively, in ErbB2 mammary epithelial tumor cell lines with different p53 status with and without treatment with Nek2 inhibitor JH295. *n* = 3 independent experiments per genotype (one cell line per genotype except for p53+/+ and p53 H/− where 2 different cell lines derived from different tumors and result per genotype was averaged). **i** Analysis of LOH in H/+;ErbB2 cell line. *n* = 3 independent experiments per treatment. Non-irradiated cells (lanes 1–3) and cells treated with JH295 (lanes 4–6) are showing no LOH. Irradiated cells showing LOH (lanes 7–9). Cells irradiated and treated with JH295 are showing no LOH (lanes 10–12). **j** Densitometric analysis of band intensity ratio of PCR amplification product shown in **i**

Strikingly, we observed a genotype-specific inhibitory effect of JH295 in mutp53 cells with p53LOH (H/−;ErbB2) as compared to cells with wtp53 allele (+/+;ErbB2, −/+;ErbB2, H/+ErbB2) as indicated by the colony formation assay (Fig. 5a, b). JH295 had an intermediate inhibitory effect on H/+ErbB2 cells (Fig. 5b). The specificity of JH295 was validated on cells where Nek2 was deleted using CRISPR/Cas9. Consistent with the requirement of Nek2 for the survival of mutp53 cancer cells, we were able to generate H/+;ErbB2/Nek2−/−, but not H/−;ErbB2/Nek2−/− cell lines by CRISPR/Cas9 technology. However, the genetic depletion of Nek2 significantly reduced the proliferation rate of H/+;ErbB2 cells in short-term assay (Fig. 5c). The analysis of mitotic H/+;ErbB2/Nek2−/− cells revealed that the genetic ablation of Nek2 did not increase the proportion of cells with centrosome amplification (Fig. 5d), but dramatically reduced centrosome clustering (Fig. 5e) with a concomitant increase in cells carrying multipolar mitotic spindle (Fig. 5f). Consistent with the genetic depletion of Nek2, the sensitivity to JH295 correlates with the complete abrogation of centrosome clustering in H/+;ErbB2 and H/−;ErbB2 cells (Fig. 5h), while the proportion of mitotic cells carrying supernumerary centrosomes did not change (Fig. 5g). Importantly, JH295 most robustly affected H/−;ErbB2 cells, but not +/+;ErbB2 cells in any tested assays (Fig. 5a, g, h), suggesting an alternative Nek2-independent mechanism of centrosome regulation in wtp53 cells. In sum, our data identified the requisite function of Nek2 for centrosome clustering and, thus, survival of H/−;ErbB2 cells.

The increased sensitivity of H/−;ErbB2 cells to Nek2 inhibition set us to test whether JH295 prevents outgrowth mutp53 cells with p53LOH, thus preventing loss of wtp53 allele after irradiation. Hence, H/+;ErbB2 cells were irradiated (9 Gy), or not, and then treated them with JH295, or not, for 10 days. DNA from surviving cells was analyzed for p53LOH by PCR. As shown in Fig. 5i, j, we observed p53LOH only in irradiated cells (lanes 7–9), but not in non-irradiated (lanes 1–3) or JH295-treated cells (lanes 4–6). Remarkably, Nek2 inhibition protects cells from irradiation-induced p53LOH (lanes 10–12).

In sum, our results suggest that Nek2 inhibition may alter the selective pressure for p53LOH in heterogeneous tumor population by contraction of specifically mutp53 population with p53LOH, thus, preventing the outgrowth of genetically unstable and metastatic cells.

## Discussion

Monoallelic mutations in the TP53 gene are widespread at the early stages of Her2-positive breast cancer (DCIS and stage 1) and usually followed by loss of the remaining wtp53 allele during tumor progression. Although previous studies suggested that mutp53 inactivates wtp53 protein in heterozygous tumors in the dominant-negative fashion [44], integrated large scale human data analysis (TCGA) argues for the strong selection for the loss wtp53 allele in tumors with monoallelic p53 mutations [45]. However, the underlying selective force for p53LOH, its mechanism, and oncogenic outcomes remain elusive.

Using MMTV;ErbB2 model carrying a heterozygous R172H p53 mutation previously, we demonstrated a novel oncogenic activity of mutp53: the exacerbation of p53LOH after γ-irradiation. We found that wtp53 partially retains the transcriptional activity allele and enables the maintenance of the genomic integrity under normal conditions in mutp53 heterozygous cells. Consistent with our mouse data, human cancer TCGA database analysis revealed that mutp53 tumors displayed a 2.5-fold higher rate of deletions at the frequent deletion sites of their wtp53 tumors counterparts [46]. Although in our previous study, we demonstrated that irradiation in the early stages of breast cancer facilitates the selective pressure for p53LOH, the underpinning mechanism remained unclear. These findings may have a significant clinical impact, as in the early stages of breast cancer patients with mutp53 heterozygous tumors [2], radiotherapy may potentially have adverse effects.

**Fig. 6:**
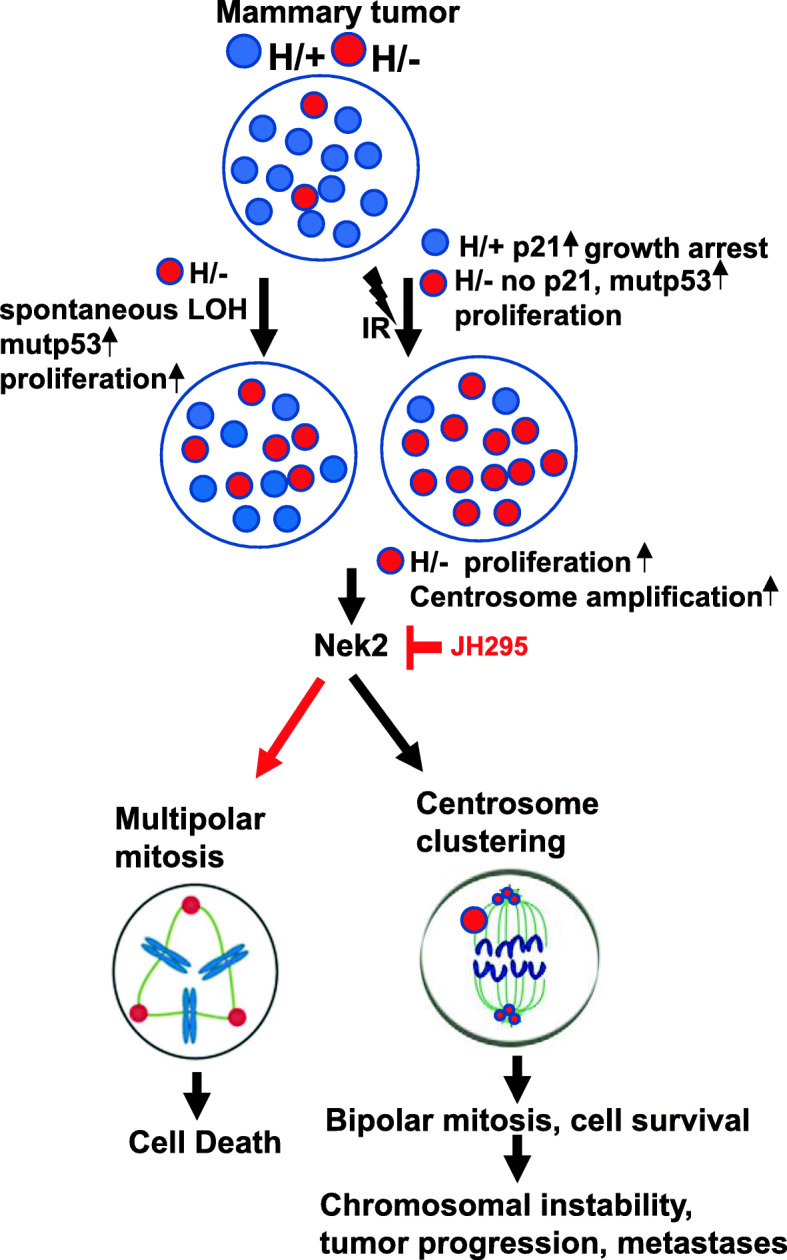
Proposed model for the role of mutp53 and Nek2 in promoting tumorigenesis. In tumors heterozygous for mutp53 there is a mixed population of heterozygous cells (H/+) and cells that underwent spontaneous LOH (H/−). Genotoxic stress, such as γ-irradiation, leads to slow proliferation and expansion of H/+ population due to the presence of wtp53 that can induce cell cycle checkpoint and arrest. On the other hand, H/− cells continue unrestricted proliferation, taking over the H/+ population. In both cases, absence of wtp53 in H/− leads to increased cell proliferation and to centrosome amplification. To avoid multipolar mitosis and cell death of H/− cells with centrosome amplification, mutp53 utilizes Nek2 to induce centrosome clustering to promote bipolar mitosis and cell survival. Centrosome clustering process lengthens mitosis which then leads to increased chromosomal instability and thus enhancing tumor progression and metastasis. Our model proposes Nek2 as an Achilles heel, for tumor cells with mutp53, that can be used as a therapeutic target to prevent p53 LOH and cells that have lost the wtp53 alleles

By using the unique panel of isogenic and non-isogenic breast cancer cell lines with the distinct p53 deficiencies, we identified functional outcomes of p53LOH in mutp53 heterozygous cells that may underlie the selective pressure for p53LOH. First, we found that p53LOH in mutp53 heterozygous cells is the crucial event in promoting mutp53 protein stabilization (Fig. 1a), which was shown to be critical for oncogenic GOF activities of mutp53 [45]. Second, p53LOH increases cell proliferation in both loss-of-function (Fig. 1d, e) and mutp53 gain-of-function (Fig. 1c, f) manner that may cause the clonal expansion cells with p53LOH. The p53LOH-enhanced proliferation can be a consequence of the loss of wtp53-induced p21 expression (Fig. 1d, e), and mutp53-mediated upregulation of mTOR pathway [3], that together increase cancer cell fitness and provide the growth advantage over heterozygous cells retaining wtp53 allele (Fig. 5l). Third, we observed a robust increase in genomic and chromosomal instability in mutp53 cells after p53LOH that may provide the genomic plasticity to acquire secondary mutations, thus, contributing to clonal expansion of cells with p53LOH. Consistent with our results, the examination of human cancer TCGA data revealed significantly enhanced chromosomal instability in mutp53 tumors that mainly lost wtp53 allele relative to their wtp53 counterparts [46]. The enhanced chromosomal instability after p53LOH can arise from increased centrosome amplification (Fig. 3b, c) and multipolar mitotic spindle formation (Fig. 3e, h) that we observed in H/−;ErbB2 cells. However, excessive centrosome amplification can be detrimental to cell viability. As a novel mutp53 GOF pro-survival mechanism, we demonstrate that H/−;ErbB2 cells adapt to avoid mitotic catastrophe or replicative senescence by bipolar clustering centrosome, allowing pseudo-bipolar division (Fig. 3b, d) at the expense of genomic instability. Fourth, p53LOH completely abrogates G2/M checkpoint in response to irradiation in the mutp53 GOF manner (Fig. 2a, e) suggesting that γ-irradiation may further facilitate the clonal expansion of mutp53 cells with p53LOH (Fig. 2a). Together, our study provides mechanistic insights into how p53LOH provides the growth advantage to mutp53 cancer cells and outcompete heterozygous cells retaining wtp53 allele and how γ-irradiation may exacerbate the clonal expansion of genomically unstable mutp53 cells with p53LOH leading to tumor progression (Fig. 6). Therefore, the targeting of pro-survival pathways activated in cancer cells after p53LOH may impede their clonal dominance and prevent tumor progression.

In an attempt to delineate the pro-survival pathways upregulated in H/−;ErbB2 cells, we identified Nek2 as a potential GOF target of mutp53. Nek2 kinase is a crucial regulator of mitotic processes such as centrosome duplication and spindle assembly. The aberrant activity of Nek2 compromises mitotic checkpoint and centrosome duplication (reviewed in [32]). Nek2 overexpression induced centrosome amplification, while Nek2 silencing decreased cell proliferation in vitro and in vivo (reviewed in [32]). Nek2 is overexpressed in various human cancers, including Her2-positive breast cancer [47], and several mutations in breast and stomach cancers have been identified (https://cancer.sanger.ac.uk) [48]. The relapse-free survival of patients with Nek2-overexpressing tumors was significantly worse than that of patients exhibiting low expression, regardless of breast cancer subtype. In support of mutp53-Nek2 link in human breast cancer, we found a strong correlation between mutp53 (all mutations, Metabric) and Nek2 mRNA expression compared to patients with wtp53 (Fig. 4f). Together, these data support Nek2 as an attractive therapeutic target in mutp53 breast cancer.

As previous studies demonstrated the inhibitory effect of wtp53 on Nek2 RNA expression [41], the loss of the wtp53 allele in heterozygous cells may induce Nek2 in a loss-of-function manner. Here, we demonstrated that in addition to loss-of-function, p53LOH in mutp53 heterozygous cells might upregulate Nek2 expression in mutp53 GOF fashion (Fig. 4e–g). Of note, median expression of NEK2 was significantly upregulated in patients with mutp53, regardless of the mutation type (Fig. 4h), not just in missense mutations. This may suggest that GOF might not be limited to a particular type of TP53 mutation, e.g., missense [49], and that all TP53 mutations might be equal at a certain level. This notion of equal TP53 mutations has been previously shown in the context of different p53 mutations exerting a dominant-negative effect [50].

While we utilized MMTV/ErbB2 mice as a model for breast cancer, a similar mechanism may take place in other subtypes of breast cancer. In support, clinical data demonstrated significant overexpression Nek2 in human triple-negative breast cancer (> 80% harbor p53 mutations) [51]. Furthermore, our retrospective analysis of Metabric data shows that Nek2 is significantly overexpressed in patients with mutp53, regardless of BC type and HER2 status (Fig. 4h). These results strengthen our notion that Nek2 overexpression is linked primarily to mutp53 presence. The mechanism by which mutp53 upregulates Nek2 is still unclear. Emerging evidence implies that mutp53 promotes malignant transformation by the physical recruitment of other transcription factors (TF) to the chromatin, thus rewiring the transcriptome towards oncogenic pathways [52]. As previously shown, Nek2 expression is downregulated by E2F4 transcription factor [53]. On the other hand, mutp53 and E2F4 proteins were shown to form a protein complex in tumor cells [54]. Thus, it is feasible that mutp53 can promote Nek2 expression by suppressing E2F4 transcriptional function. Alternatively, the overexpression of Nek2 in human breast cancer is commonly attributed to the amplification of region 1q32, the locus of the human Nek2 gene. Here, we demonstrated that p53LOH results in the significant increase of chromosomal aberrations (Fig. 3) that can lead to amplification of 1q32 locus. Alternatively, mutp53 may upregulate Nek2 expression through the forkhead transcription factor FoxM1, which was shown to positively regulate Nek2 [55, 56]. Mutp53 GOF was shown to occur in p53-AMPK-FOXO3a-FOXM1 signaling cascade to promote tumor survival and progression in head and neck squamous cell carcinoma [57]. On the other hand, while we believe that mutp53 upregulates the transcription of Nek2, there may be other direct effects of mutp53-Nek2 axis (e.g., protein-protein interactions) as well as indirect effects (e.g., phosphorylation of mutp53 by Nek2) that may contribute to aberrant mitosis. Whether mutp53 regulates Nek2 expression directly by regulating its transcription or indirectly is under current investigation in the lab.

Furthermore, the present study identified the novel requisite function of Nek2 in centrosome clustering. We demonstrated that the genetic depletion (Fig. 5e) and the specific pharmacological inhibition of Nek2 (Fig. 5h) alleviate centrosome clustering and increase the formation of multipolar spindles in mitotic cells (Fig. 5f). Therefore, mutp53-mediated upregulation of Nek2 can cause mitotic progression, thus offering the selective survival advantage to genomically unstable H/−;ErbB2 cells (Fig. 6). Not surprising that elevated centrosome clustering was associated with the increased sensitivity to pharmacological and genetic inactivation of Nek2 in H/−;ErbB2 cells. In stark contrast, Nek2 inhibitor does not affect the viability of p53+/+;ErbB2 cells (Fig. 5a). Most importantly, the selective sensitivity of H/−;ErbB2 cells to Nek2 inhibition opens the therapeutic opportunity to alter the clonal competition and prevent the outgrowth of mutp53 cells with p53LOH. In support of our hypothesis, we found that Nek2 inhibitor prevents p53LOH induced by irradiation (Fig. 5i, j).

It is worth mentioning that the clinical studies on the prognostic and predictive significance of TP53 mutations in breast cancers have been controversial [58–61]. For example, it was shown that chemo/radiotherapy-treated breast cancer patients (all stages combined, no hormone therapy), with mutp53 tumors have a greater probability of complete pathological response than wtp53 patients, whereas addition of hormone therapy improved the survival of wtp53 patients but not mutp53 patients [62]. However, the stratification by stage within the large dataset revealed that, in contrast to wtp53 patients, the survival benefits from radiotherapy for patients with mutp53 breast cancer is stage-dependent, where radiotherapy improved the survival of stage 2 patients but was detrimental to stage 1 patients [3]. Therefore, the response of mutp53 cancers can be extremely variable according to tumor type, stage, type of treatment, tumor environment and heterogeneity, and the presence or absence of other mutations [58, 59]. This indicates that for patients with mutp53 cancers, certain factors have to be considered for optimal therapeutic outcomes. Our data imply that some non-genotoxic therapies, e.g., drugs targeting cell cycle regulatory proteins in combination with radiotherapy can be used for the tumors with mutp53 depending on the stage and p53 heterozygosity.

In sum, our data suggest that Nek2 inhibition via selective toxicity prevents outgrowth of H/−;ErbB2 cells, hindering the expansion of cells with p53LOH (Fig. 6). We speculate that in heterogeneous tumor populations, p53LOH generates the clonal pool of genetically unstable cells prone to expand after γ-irradiation due to the loss of G2/M checkpoint and p21 expression leading to the selection mutp53/LOH cells. Following p53LOH, mutp53-mediated upregulation of Nek2 provides the competitive survival advantage to mutp53/LOH (H/−;ErbB2) over mutp53 heterozygous cells (H/+;ErbB2). As a pro-survival mechanism of escape from mitotic catastrophe after irradiation in the presence of centrosome amplification, mutp53/LOH cells adapt Nek2-mediated pseudo-bipolar mitosis and evasion of G2/M checkpoint by centrosome clustering.

## Conclusions

To our knowledge, this is the first evidence that p53LOH can be prevented pharmacologically, which can have a significant clinical impact. As several Nek2 inhibiting compounds were described in the literature (reviewed in [32]), their clinical development is in an early stage, and no clinical trials have been reported. Some Nek2 inhibitors have shown low nanomolar activity in vitro and significant antitumor activity in xenografts (reviewed in [32]). Finally, our data suggest that wtp53 cancers and normal tissues retaining wtp53 may be unresponsive to Nek2 inhibition. In support of this conclusion, the initial characterization of Nek2 knockout mice demonstrated no significant defects under normal conditions (International Mouse Phenotyping Consortium), suggesting potentially low toxicity of Nek2 inhibitors in normal tissues. Prospective studies in vivo will determine whether genetic and pharmacological inhibition of Nek2 prevents p53LOH and alleviates tumor progression.

## Data Availability

Human Metabric data analysis, of the somatic mutation profiles of 2,433 breast cancers, was done using data from a retrospective study [8]. The data is deposited and is publicly available at http://www.cbioportal.org. The analysis was done using the program and tools made available online at http://www.cbioportal.org. Other datasets used and/or analyzed during the current study are available from the corresponding author on reasonable request.
